# A certain invariance property of BLUE in a whole‐genome regression context

**DOI:** 10.1111/jbg.12378

**Published:** 2019-01-07

**Authors:** Daniel Gianola, Rohan L. Fernando, Dorian J. Garrick

**Affiliations:** ^1^ Department of Animal Science Iowa State University Ames Iowa; ^2^ Departments of Animal Sciences and Dairy Science University of Wisconsin‐Madison Madison Wisconsin; ^3^ AL Rae Centre of Genetics and Breeding Massey University Palmerston North New Zealand

## Abstract

A curious result from mixed linear models applied to genome‐wide association studies was expanded. In particular, a model in which one or more markers are considered as fixed but are allowed to contribute to the covariance structure by treating such markers as random as well was examined. The best linear unbiased estimator of marker effects is invariant with respect to whether those markers are employed in constructing a genomic relationship matrix or are ignored, provided marker effects are uncorrelated with those not being tested. Also, the implications of regarding some marker effects as fixed when, in fact, these possess a non‐trivial covariance structure with those declared as random were examined.

## 1 INTRODUCTION

In genome‐wide association (GWAS) studies (e.g., Manolio et al., 2009), an objective is to find statistical connections between molecular markers and genomic regions affecting some complex trait. A linear regression model including marker genotype codes as covariates is used, and the simplest version fits a single marker regression via ordinary least squares (OLS).

If aggregation or clustering due to familial or molecular similarity exists in the data, a better estimation approach is generalized least squares (GLS), as it poses a more general covariance structure than OLS (Aulchenko, de Köning, & Haley, 2007; Gianola, Fariello, Naya, & Schön, 2016; Hoffman, 2013; Kang et al., 2008; Listgarten et al., 2012; Yang, Zaitlen, Goddard, Visscher, & Price, 2014). One such structure, for example, results from declaring all or a subset of marker effects as random variables, for example, assuming that $\beta_{j}∼N\left(0,\sigma_{\beta }^{2}\right),j\;=\;1,2,\;\ldots ,\;p,$ with all markers in the set taken as independently and identically distributed random variables. A random effects specification induces marker‐based measures of similarity among individuals called molecular “relationship” or “kinship” matrices (G). The marker (s) evaluated for association is (are) treated as a fixed effect (s), and a test of nullity of effects on a trait is based on well‐established procedures.

Should the marker (s) being tested be included or excluded when building G? A priori, if a marker is declared random it could not be fixed and vice versa. Including the contribution of a marker to G while declaring it as fixed constitutes a form of “double counting.” When the number of markers (p) is very large and a single marker regression is used, the impact on G of including or removing the marker is tiny. Listgarten et al. (2012) suggest that markers being tested should be removed from G, followed by a concomitant re‐estimation of necessary variance components at each instance of testing. This approach is computationally taxing, especially when p is huge, as it is the case with DNA sequence data. In many situations, it may be reasonable to assume that variance component estimates are affected only mildly by including or excluding the tested marker in G. For many complex traits in animal and plant breeding, each of the numerous markers in a chip has a small effect on both mean and variance of the data distribution.

Gianola et al. (2016) showed that the best linear unbiased estimator (BLUE, also GLS) of the fixed effect of a marker (or sets of markers) examined in GWAS is invariant with respect to whether or not the marker (s) tested for association is (are) included in the construction of G, provided that variance components are assumed constant. This short communication expands on the preceding, as follows. First, we provide an expression that gives the variance–covariance matrix of the BLUE of each of the marker effects being tested using a simple adjustment. Second, it is shown that the best linear unbiased predictor (BLUP) of effects treated both as fixed and random is exactly zero, provided that no covariance exists between such effects and other marker effects treated as random in the model. Third, it is shown that if such covariance is not null, the fixed effects of a set of markers affect phenotypes through direct and indirect paths, and over and above the impact of linkage disequilibrium captured by columns of the matrix of genotype codes.

## 2 MODEL

The linear regression model (assume that nuisance location effects have been eliminated somehow) used in GWAS is often posed as

(1) y=Xβ+e

where y is an n×1 vector of phenotypes, X is an n×p matrix of marker genotype codes, β is a vector of p allelic substitution effects and $e\;∼\;N\left(0,I\sigma_{e}^{2}\right)$ is a residual vector where $\sigma_{e}^{2}$ is the variance of the distribution of model residuals. Let $X\;=\;\left[\begin{array}{cc} X_{1} & X_{2} \end{array}\right],$ where $X_{1}$ is $n\;\times \;p_{1}$ (without loss of generality assume that $X_{1}$ has full column rank), and $X_{2}$ is $n\;\times \;p_{2}$ where $p_{2}$ may be much larger than n.

An equivalent representation of (1) is

(2) $$y=X_{1}\beta_{1}+X_{2}\beta_{2}+e,$$

and consider two alternative covariance structures for the phenotypes. The first structure results from treating $\beta_{1}$ as a fixed vector and assuming $\beta_{2}\;∼\;N\left(0,I\sigma_{\beta }^{2}\right)$:

(3) $$V_{2}=X_{2}X_{2}^{\prime }\sigma_{\beta }^{2}+I\sigma_{e}^{2}.$$

The GLS estimate of the fixed effect $\beta_{1}$ under $V_{2}$ is

(4) $${\hat{\beta }}_{1}={\left(X_{1}^{\prime }V_{2}^{-1}X_{1}\right)}^{-1}X_{1}^{\prime }V_{2}^{-1}y,$$

The second covariance structure stems from treating $\beta_{1}$ as random, with the $\beta \;∼\;N\left(0,I\sigma_{\beta }^{2}\right)$ assumption assigned to all marker effects, but then $\beta_{1}$ is estimated as if it were fixed. Here, the phenotypic covariance matrix is

(5) $$V_{12}=X_{1}X_{1}^{\prime }\sigma_{\beta }^{2}+X_{2}X_{2}^{\prime }\sigma_{\beta }^{2}+I\sigma_{e}^{2},$$

with the GLS estimator of α, the fixed effect corresponding to $\beta_{1}$ being

(6) $$\hat{\alpha }={\left(X_{1}^{\prime }V_{12}^{-1}X_{1}\right)}^{-1}X_{1}^{\prime }V_{12}^{-1}y.$$

Note that $V_{2}\;=\;V_{12}-X_{1}X_{1}^{\prime }\sigma_{\beta }^{2}$. Arrays $X_{2}X_{2}^{\prime }\sigma_{\beta }^{2}\;=\;S_{2}$ and $X_{1}X_{1}^{\prime }\sigma_{\beta }^{2}+X_{2}X_{2}^{\prime }\sigma_{\beta }^{2}\;=\;S_{12}$ can be referred to as “similarity” matrices, as in Listgarten et al. (2012).

## 3 INVARIANCE PROPERTIES

### 3.1 Best linear unbiased estimation

Gianola et al. (2016) showed that (4) and (6) are identical; the proof is presented in more detail here. To show this, we employ a model representation where the effects of one or more loci with genotypes in $X_{1}$ are regarded as possessing both fixed and random effects:

(7) $$y=X_{1}\left(\alpha +\beta_{1}\right)+X_{2}\beta_{2}+{e=X}_{1}\alpha +X_{1}\beta_{1}+X_{2}\beta_{2}+e,$$

where α are the fixed effects of markers in $X_{1}$, with $\beta_{1}\;∼\;N\left(0,I\sigma_{\beta }^{2}\right)$ and $\beta_{2}\;∼\;N\left(0,I\sigma_{\beta }^{2}\right),$ as before.

Using the Sherman–Morrison–Woodbury identity (Seber & Lee, 2003),

(8) $$V_{2}^{-1}=V_{12}^{-1}+\sigma_{\beta }^{2}V_{12}^{-1}X_{1}{\left(I-\sigma_{\beta }^{2}X_{1}^{\prime }V_{12}^{-1}X_{1}\right)}^{-1}X_{1}^{\prime }V_{12}^{-1}.$$

Using (8), $X_{1}^{\prime }V_{2}^{-1}$ can be written as:

(9) $$\begin{array}{cc} X_{1}^{\prime }V_{2}^{-1} & =X_{1}^{\prime }V_{12}^{-1}+\sigma_{\beta }^{2}X_{1}^{\prime }V_{12}^{-1}X_{1}{\left(I-\sigma_{\beta }^{2}X_{1}^{\prime }V_{12}^{-1}X_{1}\right)}^{-1}X_{1}^{\prime }V_{12}^{-1}\\ & =\left[I+\sigma_{\beta }^{2}X_{1}^{\prime }V_{12}^{-1}X_{1}{\left(I-\sigma_{\beta }^{2}X_{1}^{\prime }V_{12}^{-1}X_{1}\right)}^{-1}\right]X_{1}^{\prime }V_{12}^{-1}\\ & =\left[I-\sigma_{\beta }^{2}X_{1}^{\prime }V_{12}^{-1}X_{1}+\sigma_{\beta }^{2}X_{1}^{\prime }V_{12}^{-1}X_{1}\right]{\left(I-\sigma_{\beta }^{2}X_{1}^{\prime }V_{12}^{-1}X_{1}\right)}^{-1}X_{1}^{\prime }V_{12}^{-1}\\ & ={\left(I-\sigma_{\beta }^{2}X_{1}^{\prime }V_{12}^{-1}X_{1}\right)}^{-1}X_{1}^{\prime }V_{12}^{-1}. \end{array}$$

Now, using (9),

(10) $$X_{1}^{\prime }V_{2}^{-1}X_{1}={\left(I-\sigma_{\beta }^{2}X_{1}^{\prime }V_{12}^{-1}X_{1}\right)}^{-1}X_{1}^{\prime }V_{12}^{-1}X_{1},$$

and $X^{\prime }V_{2}^{-1}y$ is

(11) $$X^{\prime }V_{2}^{-1}y={\left(I-\sigma_{\beta }^{2}X_{1}^{\prime }V_{12}^{-1}X_{1}\right)}^{-1}X_{1}^{\prime }V_{12}^{-1}y.$$

From (10) and (11), the GLS estimator $\hat{\beta_{1}}$ given in (4) is formed as

(12) $$\begin{array}{cc} \hat{\beta_{1}} & ={\left(X_{1}^{\prime }V_{2}^{-1}X_{1}\right)}^{-1}X_{1}^{\prime }V_{2}^{-1}y\\ & ={\left[{\left(I-\sigma_{\beta }^{2}X_{1}^{\prime }V_{12}^{-1}X_{1}\right)}^{-1}X_{1}^{\prime }V_{12}^{-1}X_{1}\right]}^{-1}{\left(I-\sigma_{\beta }^{2}X_{1}^{\prime }V_{12}^{-1}X_{1}\right)}^{-1}X_{1}^{\prime }V_{12}^{-1}y\\ & ={\left(X_{1}^{\prime }V_{12}^{-1}X_{1}\right)}^{-1}X_{1}^{\prime }V_{12}^{-1}y\\ & =\hat{\alpha }, \end{array}$$

where $\hat{\alpha },$ also given in (6), is the GLS estimator resulting from (7).

The preceding shows that the estimator obtained under covariance structure $V_{2}$ is identical to the estimator resulting from structure $V_{12}$. The practical implication is that the same similarity matrix, $S_{12}$, can be used for conducting either single marker or sets of markers GWAS studies using linear regression models, provided that $\sigma_{\beta }^{2}$ is assumed known and kept constant (as well as the residual variance) throughout.

Note that the sampling variance–covariance matrix of the estimates of the fixed effects must be taken under $V_{2}$, that is, $Var\hat{\left(\beta_{1}\right)}\;=\;{\left(X_{1}^{\prime }V_{2}^{-1}X_{1}\right)}^{-1}$. Since $X_{1}$ typically has one or a few columns in GWAS, advantage can be taken of (8) for computing $Var\hat{\beta_{1})}$, as $V_{12}^{-1}$ is obtained only once, whereas $V_{2}^{-1}$ changes with the set of markers included in $X_{1}$ and, therefore, in $X_{2}.$ Further, note from (9) that

(13) $$\begin{array}{cc} Var\hat{\left(\beta_{1}\right)}=\; & {\left(X_{1}^{\prime }V_{2}^{-1}X_{1}\right)}^{-1}={\left[{\left(I-\sigma_{\beta }^{2}X_{1}^{\prime }V_{12}^{-1}X_{1}\right)}^{-1}X_{1}^{\prime }V_{12}^{-1}X_{1}\right]}^{-1}\\ & ={\left(X_{1}^{\prime }V_{12}^{-1}X_{1}\right)}^{-1}-I\sigma_{\beta }^{2}, \end{array}$$

so $V_{12}^{-1}$ can be used throughout. The preceding representation illustrates the over‐statement of uncertainty and “loss of power” incurred by use of ${\left(X_{1}^{\prime }V_{12}^{-1}X_{1}\right)}^{-1}$ instead of (13). Yang et al. (2014) present a related discussion and recommend that markers in close linkage disequilibrium with the target marker(s) be removed when building G. Their approach requires re‐estimation of variance components at every instance of testing. Our results do not apply under such strategy, as the variance–covariance structure would be expected to change over markers tested (Listgarten et al., 2012; Yang et al., 2014). An alternative could be to include the marker tested and some neighbours in close linkage disequilibrium in $X_{1},$ provided that $p_{1}\;<\;n$ and that no rank deficiency accrues, and then use all markers when building G. A disadvantage of the alternative is the potentially strong collinearity in the set of markers in $X_{1}$, producing unstable estimates with large sampling variances.

A caveat must be mentioned. In a genomic best linear unbiased prediction setting (e.g., Legarra, 2016; Van Raden, 2008), a similarity matrix under $V_{12}$ can be constructed as

(14) $$\begin{array}{cc} S_{12} & =\frac{1}{p}\left(X_{1}X_{1}^{\prime }+X_{2}X_{2}^{\prime }\right)p\sigma_{\beta }^{2}\\ & =G_{12}\sigma_{g}^{2}, \end{array}$$

where $\sigma_{g}^{2}\;=\;p\sigma_{\beta }^{2}$ is the “genomic variance” captured by all available markers; $G_{12}$ is known as the genomic relationship matrix (Van Raden, 2008). Accordingly,

(15) $$\begin{array}{cc} S_{2} & =\frac{1}{p_{2}}\left(X_{2}X_{2}^{\prime }\right)p_{2}\sigma_{\beta }^{2}\\ & =G_{2}\sigma_{g^{\prime }}^{2}, \end{array}$$

where $\sigma_{g^{\prime }}^{2}\;=\;p_{2}\sigma_{\beta }^{2}$ is the genomic variance marked by the variants included in $X_{2}$. Clearly, two maximum‐likelihood analyses of variance components, one with a set markers fixed and removed from the covariance structure, and the other one with all markers contributing to similarity, will produce different estimates and interpretations of genomic variance.

Estimates of $\sigma_{\beta }^{2}$ must always be interpreted with great care. In a standard random effects model, the variance among effects of levels of a random factor represents a population parameter, with maximum‐likelihood estimates of variance components interpreted accordingly. For example, if the random factor is the effect of a paternal half‐sib family (a situation known as a “sire” model in animal breeding), the variance among sires, $\sigma_{s}^{2},$ say, has the same interpretation irrespective of whether the number of families is 10 or 10,000. However, in a marker‐based model with n<p, the meaning and estimates of $\sigma_{\beta }^{2}$ depend crucially on p, as the variance component acts then as a regularization parameter. In the n<p situation, it is typically the case that estimates of $\sigma_{\beta }^{2}$ decrease as p increases, and the rate of decrease in $\sigma_{\beta }^{2}$ is critical for interpretation of estimates of marker effects when p→∞ (Gianola, 2013; León‐Novelo & Casella, 2012).

### 3.2 Best linear unbiased prediction

The best linear unbiased predictor of $\beta_{1}$ in model (7) is

(16) $$\begin{array}{cc} {\vec{\beta }}_{1} & =Cov\left(\beta_{1},y^{\prime }\right)V_{12}^{-1}\left(y-X_{1}\hat{\alpha }\right)\\ & =\sigma_{\beta }^{2}X_{1}^{\prime }V_{12}^{-1}\left(y-X_{1}\hat{\alpha }\right)\\ & =0 \end{array}$$

with $\hat{\alpha }\;=\;{\hat{\beta }}_{1}$ calculated as in (4) or (6). The previous result follows because $X_{1}V_{12}^{-1}X_{1}\hat{\alpha }\;=\;X_{1}V_{12}^{-1}y$ are the GLS equations, implying that $X_{1}^{\prime }V_{12}^{-1}\left(y-X_{1}\hat{\alpha }\right)\;=\;0$.

Henderson's mixed model equations (MME) can be employed to verify result (16). For model (7) the MME are as follows:

(17) $$\left[\begin{array}{ccc} X_{1}^{\prime }X_{1} & X_{1}^{\prime }X_{1} & X_{1}^{\prime }X_{2}\\ X_{1}^{\prime }X_{1} & X_{1}^{\prime }X_{1}+I\lambda & X_{1}^{\prime }X_{2}\\ X_{2}^{\prime }X_{1} & X_{2}^{\prime }X_{1} & X_{2}^{\prime }X_{2}+I\lambda \end{array}\right]\left[\begin{array}{c} \hat{\alpha }\\ {\vec{\beta }}_{1}\\ {\vec{\beta }}_{2} \end{array}\right]=\left[\begin{array}{c} X_{1}^{\prime }y\\ X_{1}^{\prime }y\\ X_{12}^{\prime }y \end{array}\right],$$

where $\lambda \;=\;\frac{\sigma_{e}^{2}}{\sigma_{\beta }^{2}}$. Subtracting the equations for $\hat{\alpha }$ from the equations for ${\vec{\beta }}_{1}$ gives $\left(I\lambda \right){\vec{\beta }}_{1}\;=\;0$, implying that ${\vec{\beta }}_{1}\;=\;0,$ which verifies (16). Thus, solutions for $\hat{\alpha }$ and ${\vec{\beta }}_{2}$ from (17) are identical to those from the MME corresponding to a model where $X_{1}$ is excluded from forming a similarity matrix. Such model is

(18) $$y=X_{1}\alpha +X_{2}\beta_{2}+e.$$

The result ${\vec{\beta }}_{1}\;=\;0$ is easy to verify empirically (a reviewer pointed out that it is probably well known by scientists working in genetic evaluation computations) but, to our knowledge, has not been reported in the literature. A “mechanistic” explanation for (16) is that BLUP of random effects with null means depends on y through error contrasts that have a null mean vector. So, if a locus is included in a model as a fixed effect, the error contrasts used for BLUP do not possess any information on the effects at such locus. Thus, if any factor (e.g., a marker locus) is included in the model both as fixed and random, the BLUP of the random effect will depend entirely on the “prior” (Bayesian view), and, as shown above, it will be identically equal to 0.

### 3.3 Interdependent sets of marker effects

All elements of β were assumed independent and identically distributed, but the result holds for more complex dependency structures. Markers included in $X_{1}$ may be in linkage disequilibrium with those in $X_{2},$ and the phenomenon is encoded by correlations between columns of $X\;=\;\left[\begin{array}{cc} X_{1} & X_{2} \end{array}\right]$. Further, models have been suggested that include dependencies among marker effects (e.g., Gianola, Pérez‐Enciso, & Toro, 2003).

Suppose that β∼(0,B) and e∼(0,R) are independently distributed, that $\beta_{1}\;∼\;\left(0,B_{11}\right),$ $\beta_{2}\;∼\;\left(0,B_{22}\right)$ where $B_{11}$ and $B_{22}$ are non‐singular and assume $Cov\left(\beta_{1},\beta_{2}^{\prime }\right)\;=\;0$. Here, the MME equations for the situation in which $\beta_{1}$ is treated as both fixed and random take the form

(19) $$\left[\begin{array}{ccc} X_{1}^{\prime }R^{-1}X_{1} & X_{1}^{\prime }R^{-1}X_{1} & X_{1}^{\prime }R^{-1}X_{2}\\ X_{1}^{\prime }R^{-1}X_{1} & X_{1}^{\prime }R^{-1}X_{1}+B^{11} & X_{1}^{\prime }R^{-1}X_{2}\\ X_{2}^{\prime }R^{-1}X_{1} & X_{2}^{\prime }R^{-1}X_{1} & X_{2}^{\prime }R^{-1}X_{2}+B^{22} \end{array}\right]\left[\begin{array}{c} \hat{\alpha }\\ {\vec{\beta }}_{1}\\ {\vec{\beta }}_{2} \end{array}\right]=\left[\begin{array}{c} X_{1}^{\prime }R^{-1}y\\ X_{1}^{\prime }R^{-1}y\\ X_{2}^{\prime }R^{-1}y \end{array}\right]$$

Subtracting the $\hat{\alpha }$ from the ${\vec{\beta }}_{1}$ equations gives $B^{11}\;\vec{\;}\beta_{1}\;=\;0$, implying that the MME equations reduce to

(20) $$\left[\begin{array}{cc} X_{1}^{\prime }R^{-1}X_{1} & X_{1}^{\prime }R^{-1}X_{2}\\ X_{2}^{\prime }R^{-1}X_{1} & X_{2}^{\prime }R^{-1}X_{2}+B^{22} \end{array}\right]\left[\begin{array}{c} \hat{\alpha }\\ {\vec{\beta }}_{2} \end{array}\right]=\left[\begin{array}{c} X_{1}^{\prime }R^{-1}y\\ X_{2}^{\prime }R^{-1}y \end{array}\right],$$

which provide GLS(α) and $BLUP(\beta_{2})$ under a model where $\beta_{1}$ is fixed and $\beta_{2}$ is random. Note that, within block, marker effects can be correlated or uncorrelated.

Allow now for a covariance structure between the two sets of random effects, and let $Cov\left(\beta_{1},\beta_{2}^{\prime }\right)\;=\;B_{12}.$ The mixed model equations where $\beta_{1}$ is treated as both fixed and random become

(21) $$\left[\begin{array}{ccc} X_{1}^{\prime }R^{-1}X_{1} & X_{1}^{\prime }R^{-1}X_{1} & X_{1}^{\prime }R^{-1}X_{2}\\ X_{1}^{\prime }R^{-1}X_{1} & X_{1}^{\prime }R^{-1}X_{1}+B^{11} & X_{1}^{\prime }R^{-1}X_{2}+B^{12}\\ X_{2}^{\prime }R^{-1}X_{1} & X_{2}^{\prime }R^{-1}X_{1}+B^{21} & X_{2}^{\prime }R^{-1}X_{2}+B^{22} \end{array}\right]\left[\begin{array}{c} \hat{\alpha }\\ {\vec{\beta }}_{1}\\ {\vec{\beta }}_{2} \end{array}\right]=\left[\begin{array}{c} X_{1}^{\prime }R^{-1}y\\ X_{1}^{\prime }R^{-1}y\\ X_{2}^{\prime }R^{-1}y \end{array}\right].$$

Subtracting the α from the $\beta_{1}$ equations produces ${\vec{\beta }}_{1}\;=\;-B^{12}{\vec{\beta }}_{2}$, which is not null unless $B^{12}\;=\;0,$ contradicting the model assumption. Hence, when $Cov\left(\beta_{1},\beta_{2}^{\prime }\right)\neq 0$ use of $V_{2}$ or $V_{12}$ produces distinct sets of generalized least‐squares solutions, so the result for the independence case does not hold here.

If two random vectors are not independent, fixing the value of one such vector (Listgarten et al., 2012, call this “conditioning”) must alter the distribution of the other vector. Under multivariate normality, one can write $\beta_{2}\;=\;B_{21}\beta_{1}+\delta$, where $\delta \;∼\;N\left({0,B}_{2.1}\;=\;B_{22}-B_{21}B_{11}^{-1}B_{12}\right)$. The model under fixed $\beta_{1}$ becomes

(22) $$\begin{array}{cc} y= & X_{1}\beta_{1}+X_{2}\left(B_{21}\beta_{1}+\delta \right)+e\\ & =\left(X_{1}+X_{2}B_{21}\right)\beta_{1}+X_{2}\delta +e\\ & =X_{1}^{*}\beta_{1}+X_{2}\delta +e, \end{array}$$

where $X_{1}^{*}\;=\;X_{1}+X_{2}B_{21}$. Under this specification, the phenotypic covariance matrix is $V_{2}^{*}\;=\;X_{2}B_{2.1}X_{2}^{\prime }\;+\;R$ and $GLS(\beta_{1})$ should be computed as

(23) $$\tilde{\tilde{\beta_{1}}}={\left(X_{1}^{*\prime }V_{2}^{*-1}X_{1}^{*}\right)}^{-1}X_{1}^{*\prime }V_{2}^{*-1}y.$$

Likewise,

(24) $$\begin{array}{cc} BLUP\left(\delta \right) & \;=Cov\left({\delta ,y}^{\prime }\right)V_{2}^{*-1}\left(y-X_{1}^{*}\tilde{\alpha }\right)\\ & =\left(B_{22}-B_{21}B_{11}^{-1}B_{12}\right)X_{2}^{\prime }V_{2}^{*-1}\left(y-X_{1}^{*}\tilde{\alpha }\right), \end{array}$$

will predict the effect of markers in $X_{2}$ on phenotypes, conditionally on the effects of markers in $X_{1}$, that is, in the absence of genetic variation at loci in marker set 1.

Note in (22) that $X_{1}^{*}\beta_{1}\;=\;X_{1}\beta_{1}+X_{2}B_{21}\beta_{1}$ so that the “total signal” on the trait contributed by $\beta_{1}$ is decomposed into a “direct” component $X_{1}\beta_{1}$ and an indirect contribution $X_{2}B_{21}\beta_{1}$ mediated through the covariance between $\beta_{1}$ and $\beta_{2}$ $\left(B_{12}\right)$. This sort of phenomenon is well known in structural equation modelling and path analysis (Wright, 1921).

## 4 CONCLUSION

When conducting a GWAS with either a single marker or a set of markers treated as fixed, it is unnecessary to reconstruct the phenotypic variance–covariance matrix at each specific instance of testing, provided that a BLUP model is used and that marker effects in sets regarded as both fixed and random are independent across sets but not necessarily within sets. BLUE is invariant with respect to whether the genotypes of markers being tested in GWAS are employed in the construction of a genetic similarity matrix. Likewise, BLUP of the effects of the sets treated as random is invariant as well. However, the variance–covariance matrix of the GLS estimator and the prediction error‐covariance matrix must be taken with respect to the assumptions made in the model employed for analysis. If marker effects in the two subsets of genotypes have a between‐set non‐trivial dependency structure, the GWAS model requires modification.

The results presented in this paper, shown first by Gianola et al. (2016) just for GLS (BLUE), are seemingly unrecognized in the GWAS literature (e.g., Chen, Steibel, & Tempelman, 2017). An additional and probably useful result reported here is that represented by Equation (13): the variance of the estimate of any of the marker effects tested in GWAS can be obtained via a simple adjustment of the variance obtained with all markers entering into the similarity matrix.
